# Whole Genome Sequencing for Surveillance of Diphtheria in Low Incidence Settings

**DOI:** 10.3389/fpubh.2019.00235

**Published:** 2019-08-21

**Authors:** Helena M. B. Seth-Smith, Adrian Egli

**Affiliations:** ^1^Division of Clinical Bacteriology and Mycology, University Hospital Basel, Basel, Switzerland; ^2^Applied Microbiology Research, Department of Biomedicine, University of Basel, Basel, Switzerland; ^3^SIB Swiss Institute of Bioinformatics, Basel, Switzerland

**Keywords:** *Corynebacterium diphtheriae*, diphtheria, surveillance, public health, whole genome sequencing, molecular epidemiology, toxin

## Abstract

*Corynebacterium diphtheriae* (*C. diphtheriae*) is a relatively rare pathogen in most Western countries. While toxin producing strains can cause pharyngeal diphtheria with potentially fatal outcomes, the more common presentation is wound infections. The diphtheria toxin is encoded on a prophage and can also be carried by *Corynebacterium ulcerans* and *Corynebacterium pseudotuberculosis*. Currently, across Europe, infections are mainly diagnosed in travelers and refugees from regions where diphtheria is more endemic, patients from urban areas with poor hygiene, and intravenous drug users. About half of the cases are non-toxin producing isolates. Rapid identification of the bacterial pathogen and toxin production is a critical element of patient and outbreak management. Beside the immediate clinical management of the patient, public health agencies should be informed of toxigenic *C. diphtheriae* diagnoses as soon as possible. The collection of case-related epidemiological data from the patient is often challenging due to language barriers and social circumstances. However, information on patient contacts, vaccine status and travel/refugee route, where appropriate, is critical, and should be documented. In addition, isolates should be characterized using high resolution typing, in order to identify transmissions and outbreaks. In recent years, whole genome sequencing (WGS) has become the gold standard of high-resolution typing methods, allowing detailed investigations of pathogen transmissions. De-centralized sequencing strategies with redundancy in sequencing capacities, followed by data exchange may be a valuable future option, especially since WGS becomes more available and portable. In this context, the sharing of sequence data, using public available platforms, is essential. A close interaction between microbiology laboratories, treating physicians, refugee centers, social workers, and public health officials is a key element in successful management of suspected outbreaks. Analyzing bacterial isolates at reference centers may further help to provide more specialized microbiological techniques and to standardize information, but this is also more time consuming during an outbreak. Centralized communication strategies between public health agencies and laboratories helps considerably in establishing and coordinating effective surveillance and infection control. We review the current literature on high-resolution typing of *C. diphtheriae* and share our own experience with the coordination of a Swiss-German outbreak.

## Introduction

In recent years, rare but hypervirulent pathogens have been increasingly reported in specific geographic regions (1, 2), often associated with refugees and asylum seekers (3), but also in other high-risk populations including hospitalized patients (4, 5), the elderly, and newborns (6, 7). Reports of infections in refugees over the past decade have included *Borrelia recurrentis* (8), methicillin resistant *Staphylococcus aureus* (*S. aureus)* (MRSA) (9, 10), and toxigenic *Corynebacterium diphtheriae* (11). In 2016, the European Center for Disease Control (ECDC) warned about increased rates of cutaneous *C. diphtheriae* infections in Europe due to the refugee crisis (12). This pathogen came back into the focus of attention as it is (i) associated with severe infections in humans, including respiratory diphtheria (13–15); (ii) highly transmittable, indicated by the basis reproduction number with mean 7.2 (16); and (iii) known to cause larger outbreaks (17–19). For nearly two decades, in most high-income countries, cases have been reported rarely, occasionally in travel returners (20–24), drug users and homeless people (25–29). In the last few years, in contrast, cutaneous, and respiratory infections have predominantly been reported in refugees (16, 30–38).

Providing state-of-the-art diagnostics for rare and unexpected pathogens can be a challenge for the clinician (39) and the routine microbiology laboratory (40–42). Often specific diagnostic tests are only available in reference laboratories, thus further delaying efficient therapy, surveillance reporting, and outbreak management. Once the pathogen is cultured and identified, molecular typing technologies, such as whole genome sequencing (WGS), allow a detailed comparison on the genomic level with high resolution (43–45). In the case of *C. diphtheriae*, high-resolution typing is helpful to (i) provide the epidemiological broader context (35) and (ii) include or exclude transmission events between patients (30, 31).

WGS specifically, gives the highest resolution typing, and can help to identify potential sources and transmission routes as part of modern surveillance technologies. Recent comparisons using WGS data analyzed by core genome MLST (cgMLST) or single nucleotide polymorphisms (SNP)-based methods have shown significant improvements over older technologies (46, 47). The advantages of using WGS for high-resolution typing has been seen in several pathogens, being particularly helpful in settings with (i) highly similar isolates over a long time period e.g., *Legionella pneumophila* within a city (48) or *C. difficile* (49, 50), (ii) a low endemic epidemiological background, but multiple clusters of patients from high endemic region with potential transmission events e.g., *C. diphtheriae* (31) or *M. tuberculosis* (51), and (iii) high endemic burden, where transmission events cannot easily be separated based on classical epidemiological information alone.

Alongside the availability of rapid diagnostic tests and high-resolution typing, surveillance programs are an important cornerstone of public health, as the associated framework allows data collection, communication, and coordination of public health interventions. Of note, to date no global or European surveillance network exists which integrates both classical and molecular epidemiological data into a single real-time updated platform. Future surveillance programs may not only incorporate baseline features of an isolate such as sequence type and presence or absence of the *tox* gene, but also more detailed genomic analysis and a virulence factor profile. The aim of this would be to better assess the potential of a strain to cause outbreaks with more severe clinical phenotypes. In this review article, we will focus on *C. diphtheriae* as a re-emerging but rare pathogen, and will discuss the various aspects of classical and molecular epidemiology utilizing new sequencing technologies for surveillance.

## Microbiology and Pathogenicity of *C. diphtheriae*

*Corynebacterium diphtheriae* was first isolated in 1884 by Loeffler (52). The classical presentation is pharyngeal diphtheria, a toxin-mediated infectious disease of the upper respiratory tract. The hallmark feature is an inflamed pseudo-membrane on the pharynx, potentially causing asphyxia (13). Beside respiratory infections, *C. diphtheriae* may cause skin infections and other invasive diseases such as endocarditis, osteomyelitis, and septic arthritis (53–58). At the moment, non-toxigenic cutaneous diphtheria is the most prevalent clinical presentation (24, 39, 57, 59, 60). Wound infections often occur with other skin pathogens, such as *Streptococcus pyogenes* or *S. aureus* (28, 31). Cutaneous diphtheria may be a source of toxigenic pathogens and may be transferred to other body sites then potentially causing respiratory diphtheria. Therefore, even wound infections with non-toxigenic strains might ideally be considered to be reported to surveillance programs in order to identify carriers, clusters of potential transmissions, and high-risk groups.

### Microbiology

The species *C. diphtheriae* is divided into four biochemical biovars—belfanti, gravis, intermedius, and mitis (15, 61). Although the biochemical distinctions are not reliable, for historical reasons reference laboratories still use them. Recently, two distinct subspecies have been proposed based on genomic features: *C. diphtheriae* subsp. *diphtheriae* and *C. diphtheriae* subsp. *lausannense*. Of interest, members of the newly described subspecies *lausannense* show a larger genome size and are enriched in genes related to transport and metabolism of lipids and inorganic ion (62). On the other hand, the new subspecies lacks all genes involved in the synthesis of pili, molybdenum cofactor, and nitrate reductase. Closely related to *C. diphtheriae* are two zoonotic pathogens, *C. ulcerans* and *C. pseudotuberculosis* (63), both of which can acquire the toxin gene via a phage (64). Increasing numbers of toxigenic *C. ulcerans* infections have been reported (65, 66) e.g., in the UK (67), but these pathogens remain rare in the clinic. Host jumps from domesticated and wild animals to humans have been postulated (63, 68, 69). If either *C. ulcerans* or *C. pseudotuberculosis* is diagnosed, the isolate should be tested for the presence of the toxin and reported in surveillance programs.

### Virulence Factors

The ß-corynephage encodes the diphtheria toxin, and can be transmitted between isolates. The ß-corynephage may pose a survival benefit for the bacterium by increasing the effectiveness of transmission by helping to cause local tissue damage (14, 70). The DtxR regulator is present elsewhere in the genome, and controls the transcription of the toxin gene (*tox*). This regulator is a key determinant for iron homeostasis (71). Iron is crucial for a number of cellular functions and the expression of a toxin in situations with low iron concentrations might help pathogens to compete with the host for iron or release iron via lysis of host cells. Of particular importance are pili encoded by *spa* operons (*spaABC, spaDEF*, and *spaHIG*), which contribute to the interaction with the host. Gain or loss of the function of these genes correlate to the number and expression of pili on the cell surface—especially the major pilin genes *spaA, spaD*, and *spaH*. The *spaA*- *spaD*- and *spaH*-type pili interact with the pharyngeal, laryngeal, and lung epithelial cell types, respectively (72). Pilus expression may strongly influence the virulence of a strain (73–78), especially in combination with the presence of the *tox* gene.

### Diagnostic Aspects

Specific culture media such as tellurite agar improves the culture of *C. diphtheriae* (61, 79)—although the agar adds some selection, most diagnostic laboratories do not carry the agar as part of routine stock. The three species of interest, *C. diphtheriae, C. ulcerans*, and *C. pseudotuberculosis*, can be reliable identified with matrix-assisted laser desorption ionization time-of-flight mass spectrometry (MALDI-TOF MS) (80–82). More specialized laboratories have the capacity to detect the presence of the diphtheria toxin either by PCR or measurement of toxin production by a modified Elek test (31, 83, 84). Other virulence factors such as pili are generally not determined in routine diagnostics. A survey of the diphtheria surveillance network (DIPNET) indicated that many centers were not able to isolate the target organisms, and most found difficulties differentiating them from specimens that contained *Corynebacterium striatum*, a commensal contaminant (85). More recently, an ECDC technical report on the diagnostic gaps has been published (86). Regular workshops and external quality assessments are important aspects in maintaining diagnostic quality for rare pathogens in the context of a surveillance program.

## The Return of an Old Foe

### Importance of Vaccination

In 2016 the EDSN reported 47 laboratory confirmed cases of *C. diphtheriae* and *C. ulcerans* in European countries—corresponding to an overall notification rate below 0.01 cases per 100,000 people (66). In contrast, worldwide, 7097 diphtheria cases were reported in 2016, mainly in low-income countries (www.who.it). In the 1900s−1950s, infections with *C. diphtheriae* were among the most severe infections during childhood, especially in pre-school children where case fatality rates of 2–25% were reported (87, 88). Prior to the availability of toxoid-vaccines, nearly 70% of the cases were in children younger than 15 years of age (89). With the introduction of vaccines in the 1940s and 1950s, a significant decrease in incidence was observed (87, 88, 90–93), although no controlled clinical trial to evaluate the efficacy of the toxoid-vaccines in preventing diphtheria has ever been performed.

The current WHO recommendation states that a series of three toxoid-vaccine doses should be provided, starting at six weeks of age, with additional booster doses based on local epidemiology (94). Vaccine effectiveness is high after three or more doses, ranging from 96 to 98% (95, 96). Although not assessed in routine, an antitoxin level of 0.01 IU/mL provides the lowest level of protection, 0.1 IU/mL is considered a protective level, and levels of >1.0 IU/mL result with long term protection (61). Interestingly, two cases of fatal diphtheria in patients with antitoxin levels above 30 IU/mL have been reported, suggesting that no absolute protection exists (97). Although immunization programs of infants started in the late 1970s, the vaccine coverage rates of infants in developing countries increased only slowly from 46% in 1985 to 79% in 1992 (98). If vaccines rates in the general population are too low, herd immunity fails to protect the non-vaccinated population, resulting in outbreaks with the potential for high mortality in younger and older age groups. An assessment of the immunity against a series of pathogens in adult asylum seekers in the Netherlands showed median 82% seroprotective anti-toxin titers against diphtheria (99). Although diphtheria vaccine rates in infants range from 89 to 98% in most European countries, a recent meta-analysis showed that vaccine rates against diphtheria and tetanus toxoids, and acellular pertussis (dTap) in healthcare workers was only 45.1% in the US and 63.9% in France (100). In Luxembourg only 2.5% of individuals under the age of 20 were seronegative, while 42% of individuals over the age of 40 years were seronegative (101). Similar low seroprotection rates have been documented in China, where only 34.1% of subjects older than 40 years were seroprotected (102). The reason for low seroprotection in some population groups in countries, where the vaccine is available, may result in a decrease in circulating toxigenic *C. diphtheriae* isolates (89), resulting in (i) an increase in non-toxigenic cases (103), and (ii) lower natural boost effects of antibody titers against the toxin (104). Especially in the adult population, gaps in herd immunity have been described due to waning of protective antibodies either from lower natural exposure or booster-vaccination. It has been found that the diphtheria vaccination only prevents symptomatic infection, and does not inhibit carriage or transmission of the pathogen. Miller and colleagues have shown that a high percentage of *C. diphtheriae* carriers were fully vaccinated, suggesting that antibodies against the toxin does not inhibit nasopharyngeal colonization (93). Based on this data, we may conclude that adults and the elderly are at higher risk of *C. diphtheriae* infection. Regular assessment of seroprotection rates in a given population should be a part of surveillance programs.

### Changing Epidemiology

In the 1960–1970s, any outbreaks described in high income countries were smaller (92, 105–108) in comparison to the larger outbreaks which occurred in the late 1990s and early 2000s, particularly in countries of the former Soviet Union (17–19, 109–113). A very large outbreak affected states of the former Soviet Union with more than 150,000 infected people and between 3,000 and 5,000 deaths (18). In this outbreak, a high proportion of adults were affected, potentially due to disruption of health services resulting in poor vaccine coverage (114, 115) and reduced “natural” exposure over the preceding decades, resulting in antibody titers below protective levels (116–118). In recent years, multiple outbreaks, or potential transmission clusters have been reported in: Bangladesh (119, 120), Brazil (121), Colombia (122), Germany (30, 35), India (123–125), Indonesia (126), Laos (127), Norway (128), Nigeria (129), Poland (130), Spain (38), South Africa (36, 131), Syria (132), Switzerland (31), Thailand (114), the United Kingdom (37), Venezuela (133, 134), and Yemen (135). The global list of affected countries indicates that (i) the disease is remains poorly controlled, (ii) the main burden lies in low-income countries, and (iii) local and global surveillance should be intensified in order to better control the disease.

## Epidemiology: From Classical to Molecular

Some of the key factors driving the spread of hypervirulent pathogens include poor vaccine rates, waning antibody titers, reduced access to healthcare, failing, or collapsing healthcare systems, poor hygiene, transfer of patients between healthcare institutions, changes in travel behaviors, increased traveling to high endemic regions, and migration from high endemic regions due to violent conflicts or for economic reasons (136–138). The development of effective preventative strategies to reduce the impact of hypervirulent bacteria should, as for multidrug resistant (MDR) pathogens, have a top global priority among public health experts, clinical microbiologists, and infectious diseases physicians. The basis for preventative strategies relies on two key elements: classical and molecular epidemiological data.

*Classical epidemiological methods* are used to investigate an unexpected frequency of specific pathogens clustering within a certain time and/or geographical range. Determining a case definition is an important first step. Cases have to be confirmed, background rates established, and patient data collected via, for example, structured questionnaire, and accessing detailed medical history. Thus, a hypothesis for the disease transmission can be formulated and potential sources named (139, 140). Although classical epidemiological methodologies provide tremendously important information, data collection is often challenging due to delayed or incomplete reporting of cases, lack of centralized communication strategies, especially at the beginning of an outbreak, vague medical history, language barriers, and cultural differences. Especially in the case of refugees, where classical epidemiological data are often not reliable, available or re-constructible, in many cases classical methods cannot provide the required data.

*Molecular epidemiological methods* are based on detailed comparison of pathogens, using some or all of the genomic information. The relatedness of pathogens can be visualized in trees, thereby helping to cluster isolates and provide information on potential molecular epidemiological links. Several genotyping approaches have been used for *C. diphtheriae* including ribotyping, amplified fragment length polymorphisms, PFGE, random amplified polymorphic DNA (RAPD), clustered regularly interspaced short palindromic repeat (CRISPR)-based spoligotyping and MLST (141–149). Some typing methods show better resolution than others: ribotyping outperforms PFGE and AFLP in terms of discriminatory power (143). Ribotyping was for many years considered the gold-standard before the introduction of a robust MLST approach. Many ribotypes were allocated a geographical name based on the location of the initial isolate, however some followed an arbitrary nomenclature (144). CRISPR-based spoligotyping can offer additional resolution within ribotypes, and be used successfully to further characterize outbreak-associated strains (147, 148): the epidemic strains from the former Soviet Union belonged to two ribotypes (St. Petersburg and Rossija) that could be subdivided into 45 additional spoligotypes (146, 147). Data from various outbreaks shows the relative high molecular diversity of isolates indicating that new strains are emerging regularly within this species (150).

A robust MLST scheme was developed in 2010, including the genes *atpA, dnaE, dnaK, fusA, leuA, odhA*, and *rpoB* (www.pubmlst.org/cdiphtheriae). The advantages of an MLST scheme include transferability and comparability. The sequence types were shown to be consistent with the previously determined ribotypes and offered higher resolution in most cases (141). MLST diversity has grown continuously, with 608 types currently categorized (March 2019). Of note, the MLST scheme lacks the biochemical correlation of the biovar system and STs have not been able to be associated with a more severe clinical phenotype (141, 151, 152).

Comparison of the performance of various typing techniques is important, as low resolution typing methods may overcall transmission events masking the real transmission steps and potentially delaying the identification of the source. Stucki et al. showed this for *M. tuberculosis* transmissions events in Switzerland, where a VNTR low-resolution typing gave evidence of a significantly higher rate of transmissions events in comparison to WGS based typing on the same set of isolates (153). Similarly, *C. diphtheriae* SNP-based WGS comparisons improved the typing resolution in comparison to cgMLST (35).

## Whole Genome Sequencing of *C. diphtheriae*

The first complete genome sequence of *C. diphtheriae* (strain NCTC13129) was analyzed in 2003, a UK clinical isolate containing a series of pathogenicity factors including iron-uptake systems, adhesins and fimbrial proteins (154). The genome of *C. diphtheriae* is 2.45 Mbp with a G+C content of 53.5% (154). Through WGS analysis we can determine the presence of virulence factors such as the toxin gene (and ß-corynephage) and pili, and genes encoding antimicrobial resistance determinants (62, 155, 156). During outbreak and public health investigations, WGS SNP-based typing clearly shows important benefits due to its high resolution (31). Although MLST may be more cost effective, MLST data can also be extracted from WGS data, providing the ST as well as high resolution phylogeny and additional important genetic information. WGS can identify additional toxins and adherence factors, which may allow the generation of a specific risk profile for the pathogen.

Comparative studies have shown that the species has a set of ~1,630 core genes which almost every representative of this species possesses [60% of the genome], and a relatively large, open pan-genome (155, 156). The difference in genome content across the species is largely due to the presence of genomic islands, prophages, transposons, restriction-modification systems, and CRISPR elements. Horizontal transfer substantially helps to shape the bacterial genome (62, 155). Some of the identified genomic islands carry genes for siderophore synthesis and transportation and degradation of polysaccharides, and heavy metal resistance. Interestingly, prophages are genetically more similar within specific clusters of bacterial isolates than between clusters, suggesting that prophages do not randomly mix between isolates, but rather cluster within specific clades (31, 157, 158).

While MLST analysis first suggested, that there is significant recombination within *C. diphtheriae* (141), this has been confirmed through analysis of whole genome sequences (159). Recombination plays an important role in bacterial evolution and has been linked to increased virulence in some pathogens (160–162). Especially in the upper respiratory tract, where *C. diphtheriae* can form a colonizing state, horizontal gene transfer can commonly happen (163). WGS allowed to study genetic ancestry of multiple bacterial species—including *C. diphtheriae*. This challenged sometimes our current understanding and groups based on biochemistry or serotypes may change. As an example, it has also been shown that biovars of *C. diphtheriae* do not correlate to genetic ancestry (152, 159). In recent years, several cohorts of *C. diphtheriae* isolates have been analyzed using WGS (30, 31, 35, 36, 62, 152, 155, 156, 164–167). Comparison of WGS data across a species generally uses one of two approaches: cgMLST, or SNP-based variant calling across the whole genome based on a reference, which provides more information and higher resolution. Dangel et al. have generated a cgMLST scheme including 1553 target loci and an extended cgMLST scheme including 2154 target loci, providing higher resolution (35).

**Figure 1:**
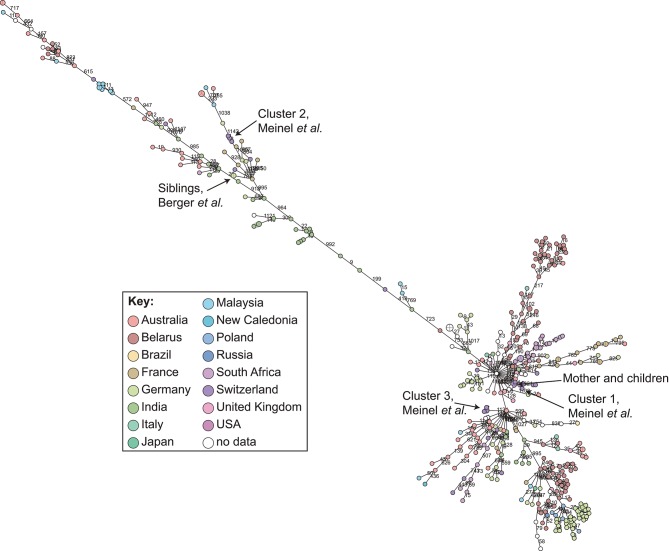
Minimum spanning tree showing relationship of all available *Corynebacterium diphtheriae* genomes by cgMLST. All *C. diphtheriae* assemblies available from NCBI on 27.02.2019 were retrieved, and compared to isolates from our laboratory, as well as all reads available from NCBI on 08.10.2018, and those published, which were assembled using unicycler (168), and duplicate samples removed, giving *n* = 419 genomes. The cgMLST scheme of (35) was used within Ridom SeqSphere+ v4.1.6, with clusters given between nodes with five or fewer differences. Nodes are colored according to country of isolation.

**Figure 2:**
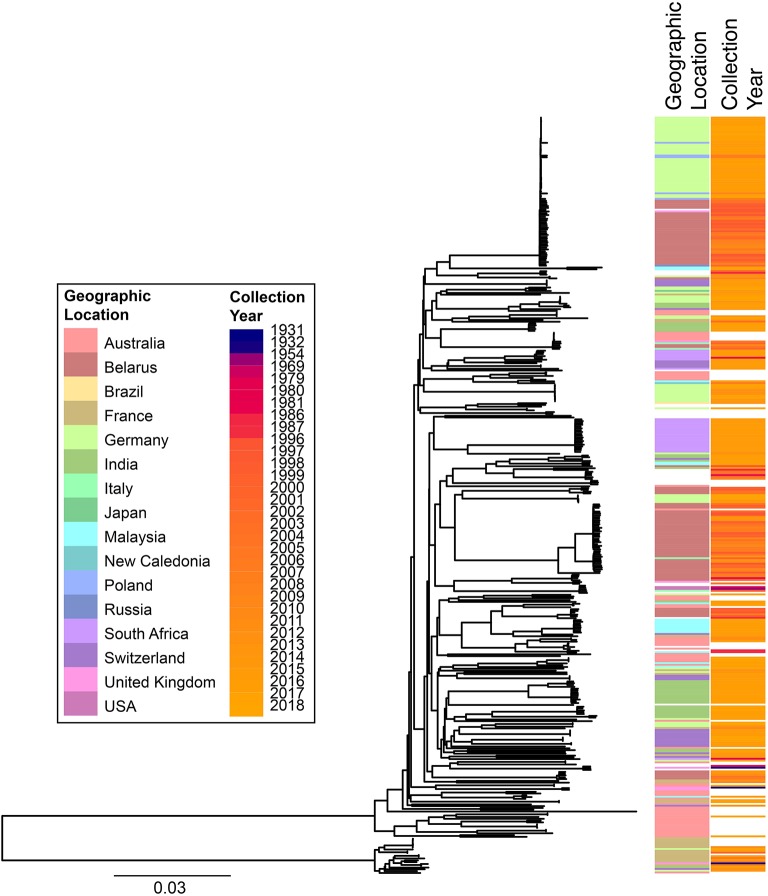
Phylogenetic overview of all available *C. diphtheriae* genomes. All *C. diphtheriae* reads available from NCBI on 08.10.2018 were retrieved, and those published, and compared to isolates from our laboratory, as well as all assemblies available from NCBI on 27.02.2019, which were shredded to reads using wgsim in samtools (https://github.com/lh3/wgsim), and duplicate samples removed, giving *n* = 419 genomes. All reads were mapped against the reference genome CP003210 (155) within CLC Genomics Workbench 10.1.1, also used to generate a single nucleotide polymorphism (SNP) phylogeny with parameters that differed from the default as: variant calling with 10x minimum coverage, 10 minimum count and 70% minimum frequency, and SNP tree creation with 10x minimum coverage, 10% minimum coverage, 0 prune distance and including multi-nucleotide variants (MNVs). Metadata was retrieved from the NCBI database and was associated with the phylogeny using phandango (169). Colors use the same key as Figure 1; years are shown on a continuous scale. The bottom clade shows the clearly separate cluster proposed as *C. diphtheriae* subsp. *lausannense*.

cgMLST and SNP-based analyses of all publicly available whole genome sequences (Figures 1, 2 and Supplementary Table 1) shows vast diversity, and geographic mixing: isolates identified in Malaysia, India, Australia, and Switzerland are found throughout the trees. Relatively few cgMLST clusters are defined at the five allele cut off, yet some clades/clusters clearly show geographic association, such as those from South Africa, Belarus and Germany (35), suggestive of local outbreaks. The largest clade of highly related isolates, at the top of Figure 2, includes those from Germany, Poland, the UK and the former Soviet Union, suggesting that these may have had a common source, but spread prior to diagnosis (This clustering is not represented in the minimum spanning tree of Figure 1). However, the dates of the isolates in this clade range from 1996 to 2017, also suggesting some stability of the isolates over time. This is also evidenced as closely related isolates throughout the tree may have been isolated many decades apart.

### Clustering and Likelihood of Transmission

There is an ongoing debate about defining diversity thresholds to separate clusters of pathogens. Determining a threshold of diversity to reliably describe a transmission cluster is a question commonly asked, yet difficult to answer, particularly in recombinogenic bacteria. Dangel et al. defined a cluster in their cgMLST scheme as five or fewer allele differences, with higher resolution of subclusters analyzed through an extended cgMLST scheme (35).

In order to determine a reliable cut-off, it is beneficial to combine the genomic analysis with more classical epidemiological data, which significantly contributes to understanding the transmission risks. However, in the literature and epidemiological data associated with WGS, few such cases have been described: in one case of direct transmission between siblings, the isolates show no allele differences in the defined core genome or accessory genome (30); and one case of direct transmission from mother to twin newborns showed a single SNP between the isolates on a whole genome level (unpublished data) and zero allele differences in the cgMLST scheme (Figure 1).

During our study on isolates from refugees in Basel, we asked ourselves if the observed whole genome diversity of 50–150 SNPs within clusters could represent a recent transmission event. We considered two different mutation rates representing extremes of plausible ranges, and estimated the approximate transmission dynamic. Even using a very high mutational rate of 0.00018 substitutions/bp/year, the estimation indicated that transmission occurred more than four to 6 weeks prior to sampling. In that paper, we played with substitution rates an picked the mutation rate of *Helicobacter pylori*, in order to have a highly conservative estimated if the transmission occurred on European ground to trigger potential outbreak investigations. This helped to exclude a transmission event within Europe, as the affected refugees arrived 2 weeks prior in South Italy (31). Analyzing these clusters by cgMLST shows that the isolates diverge by 0–4 alleles (Figure 1), within the cluster threshold, despite possessing at least 50 SNP differences and not representing recent transmission (31). This exemplifies the increased resolution of using whole genome SNP-based methods, and the difficulty of inferring direct transmissions from cgMLST data alone. As *C. diphtheriae* can also undergo recombination, it is crucial to consider a recent recombination by studying the distribution of SNPs across the genome: if many SNPs cluster in one or more genomic loci, then a recombination event is likely to have occurred, bringing the putative transmission event more recent.

## Surveillance

Although country specific surveillance systems for hypervirulent pathogens such as *C. diphtheriae* exist, the interoperability of data and the exchange across countries presents problems (170). In 2014, a WHO-recommended surveillance standard of diphtheria was published. This included a case definition, laboratory criteria for diagnosis, and minimum data elements which should be collected (171). Similarly, the ECDC has established a surveillance program for diphtheria. Founded in 1993 as European Laboratory Working Group in Diphtheria in 2006 it became the European Diphtheria Surveillance Network (EDSN, www.ecdc.europa.eu) (172). The network provides valuable information and aims to standardize surveillance activities and ensure availability of more comparable data between countries. It also includes laboratory components focusing on trainings and external quality assessments (EQAs), strengthening the laboratory capacity to characterize isolates and develop novel tools for molecular typing of *C. diphtheriae*.

While the EDSN provides an important framework for surveillance of *C. diphtheriae*, in the current refugee crisis, multi-national coordination of outbreak investigation is clearly a challenge. Rapid and effective mechanisms of communication are crucial. Patients may be evaluated several times on their journey, and the same pathogen may be isolated in different countries. A recent report on the tracing of an MDR *M. tuberculosis* cluster was very well-coordinated by a joint effort from multiple centers (51). Similarly, for *C. diphtheriae*, we directed an investigation with multiple refugees presenting with wound infection across different hospitals and diagnostic laboratories in Switzerland in 2015 (31). In both situations, a multi-national taskforce organized a coordinated effort to collect isolates and information, using case report forms to collect structured epidemiological information on migration routes, vaccine status, and other affected travelers.

Individual responsible experts, such as representatives of the EDSN or reference laboratories, should be assigned in each country in order to keep track of potential movements of refugees. In the *C. diphtheriae* situation, refugees were rapidly lost to follow-up, for example due to relocation in other refugee centers. Communication to refugee center responsible personnel and physicians should be established. The molecular epidemiology of diphtheria would certainly benefit from implementation of WGS. Such analysis offers improvements over the current model of global tracing of large clonal clusters toward fine-tuned strain discrimination. At the same time, a multicenter evaluation of recently developed inexpensive and discriminatory VNTR and CRISPR methods is warranted to see if and how they could complement regional surveillance (150). Beside the molecular definition of an outbreak, a centralized database allows running the standardized bioinformatic algorithms and thereby may provide a benefit for investigations. Isolates could be registered with particular coded identifiers to avoid re-sequencing the same isolate (173).

To date, no database can integrate classical epidemiological data in the form of coded patient identification, vaccine status, potential exposures, spatiotemporal information of cases, socioeconomic and immunological data on a population level, with high-resolution molecular epidemiological data from sequenced strains. We are developing such a platform, initially for MDR pathogens (173), which could easily be expanded to hypervirulent species including *C. diphtheriae*. This Swiss Pathogen Surveillance Platform (www.spsp.ch) aims to integrate all relevant data in the near future, thereby providing various stakeholders with important information in real-time. Such a platform may provide a public health data sharing hub not only for Switzerland, but for European countries and beyond.

### Warning Systems

In many countries, reporting of *C. diphtheriae* cases to public health authorities is mandatory. Information is collected and reported back to the diagnostic laboratories and infectious diseases specialist in order to heighten awareness. Various email alerting system for surveillance exists, one of the most well-known being PROMED (https://www.promedmail.org/), a subscription service which has been in place since the early 2000s (174). Those warning systems collect information from media reports, official reports, online summaries, local observers, subscribers, and others. However, those services rely on reporting toward the service and also inaccurate interpretation and privacy issues may be an issue. Nevertheless, there is still room for faster, more targeted and international ways of communication to be established. The connection of various data sources will require the usage of standardized and specific epidemiological ontologies being used across various databases such as SNOMED CT (www.snomed.org) or IRIDA (www.irida.ca). The ethical and legal implications of such big-data driven surveillance programs need to be clarified in the near future. Clearly individual patient data should be protected, but those rights should be balanced in situations where outbreaks with hypervirulent pathogens may put the general population at risk—in the case of *C. diphtheriae* the risk for the general healthy population in Western countries seems rather low and therefore surveillance efforts should rather focus on at-risk populations. Social media may be used to generate epidemiological data but could also be used as a tool to inform the general public and health care specialists. We could imagine internet-based warning systems being combined with a more detailed platform allowing clinicians to assess classical and molecular epidemiological aspects.

### Machine Learning for Investigation and Surveillance of Rare Pathogens

In the near future, we can foresee interconnected databases containing epidemiological data on individual cases, incidence rates of particular infections, spatiotemporal clusters, WGS data, travel and migration information, social and print media reports, and vaccine rates in populations. These may then be used for machine learning based epidemiological surveillance, such as that recently published on prediction of dengue outbreaks (175).

Machine learning based algorithms may also be used to predict the case severity of a particular infection based on NGS and other clinical data, as similar performed by Njage et al. in the case of shigatoxigenic *E. coli* (176). Bacterial genome wide association studies (GWAS) using machine learning in *C. diphtheriae* may help to identify critical biomarkers, linking bacterial genomic features such as virulence or resistance with specific host outcomes. Such work often requires hundreds to thousands of bacterial genomes to compensate for host variability effects (177) as shown for *M. tuberculosis, Campylobacter* spp. and *Bordetella* spp. (178–180).

The advances in machine learning algorithms may allow the development of revolutionary surveillance programs, potentially providing valuable information to public health policy makers about potential epidemiological trends and risks for the general public. Although such databases are likely to first be established for more common epidemic scenarios such as annual influenza, MDR pathogens, and foodborne pathogens, particular risks may also be calculated for rare pathogens such as measles, ebola, or hypervirulent bacteria such as *C. diphtheriae*. As we live in an increasingly globalized world with rapid spread of pathogens, new concepts for epidemiological surveillance are needed, to enable rapid and effective interventions.

## Conclusions

*Corynebacterium diphtheriae* is reemerging in clinics in high income countries, partly as a result of refugee movement, and requiring greater awareness of the issue. WGS offers the opportunity to describe potential transmission events and infection sources with the highest resolution. Data provided from molecular typing methods should, where possible, be analyzed in the context of classical epidemiological information, for which information has to be rapidly shared with local public health authorities. In addition, surveillance for *C. diphtheriae* and other re-emerging hypervirulent pathogens would benefit from rapid data collection and sharing platforms sharing information on classical and molecular epidemiology.

## Author Contributions

HS-S performed data analysis and wrote the manuscript. AE wrote the manuscript.

### Conflict of Interest Statement

The authors declare that the research was conducted in the absence of any commercial or financial relationships that could be construed as a potential conflict of interest.
